# Characterizing Biomarkers of Muscle Damage in Collegiate Football Players: A Prospective, Repeated Measures Study

**DOI:** 10.3390/jcm15072502

**Published:** 2026-03-25

**Authors:** Grace Brandhurst, Erik Piedy, Stephen Etheredge, Matthew Martone, Heather D. Quiriarte, Paul Phillips, Derek Calvert, Nathan P. Lemoine, Jack Marucci, Brian A. Irving, Robert Zura, Guillaume Spielmann, Neil M. Johannsen, Rachel Matthews

**Affiliations:** 1 Department of Orthopaedics, School of Medicine, Louisiana State University Health Sciences Center, New Orleans, LA 70112, USA; 2 LSU Athletics, Louisiana State University, Baton Rouge, LA 70802, USA; 3 School of Kinesiology, Louisiana State University, Baton Rouge, LA 70802, USAhquiriarte1@lsu.edu (H.D.Q.);; 4 Institute for the Health & Performance of Champions, Louisiana State University, Baton Rouge, LA 70802, USA; 5 Pennington Biomedical Research Center, Baton Rouge, LA 70808, USA

**Keywords:** creatine phosphokinase, exertional rhabdomyolysis, athletes, renal function, liver function

## Abstract

**Background/Objectives:** Exertional rhabdomyolysis (ER) is a possibly fatal condition resulting from extreme or novel exercise that causes substantial muscle breakdown. ER has been observed during preseason football; however, prospective research has yet to characterize normal versus ER responses using a repeated measures design. This study characterized ER biomarker responses related to muscle damage, and renal and hepatic stress, after two NCAA Division I preseason football scrimmages. **Methods:** Following a prospective, repeated measures design, blood and urine samples from 17 players were collected immediately (IPS) and 24 h post-scrimmage (24hPS). A subset (*n* = 13) provided samples after 48 h of rest as a non-exertion (NE) comparator group. A Comprehensive Metabolic Panel was run on serum samples, and urine samples were analyzed for myoglobin and creatinine. Values were compared with reference ranges, mixed models evaluated time effects, and linear regressions examined associations between CPK and renal and hepatic biomarkers. **Results:** No participants were diagnosed with ER. A time effect was observed for CPK (*p* < 0.01), with CPK greater IPS (991.6 ± 560.8 IU/L) compared to NE (267.7 ± 205.3 IU/L), and remaining elevated above reference ranges at 24hPS (739.2 ± 442.6 IU/L). Similar time effects were observed with LDH, AST, and ALT (*p* < 0.01). Serum creatinine increased above reference values and NE concentrations (*p* < 0.01). CPK correlated (*p* < 0.01 for all) with LDH (r = 0.69), serum myoglobin (r = 0.57), creatinine (r = 0.42), AST (r = 0.77), and ALT (r = 0.38). **Conclusions:** Biomarkers of muscle damage, renal stress, and liver function were higher IPS, with only partial recovery by 24hPS. These findings provide preliminary reference patterns for biomarker fluctuations and support individualized, serial monitoring to identify abnormal responses and promote early detection of ER.

## 1. Introduction

Athletes frequently experience muscle damage induced by intense exercise, as evidenced by elevations in biochemical markers of musculoskeletal stress [1]. Monitoring these biomarkers is essential not only for evaluating performance and recovery but also for detecting conditions, including exertional rhabdomyolysis (ER). ER refers to the breakdown and necrosis of striated muscle induced by strenuous exercise, which releases muscle cell contents into the bloodstream and, if untreated, can lead to severe, systemic complications including acute kidney failure, cardiac arrhythmia, and death [1,2]. The National Athletic Trainers’ Association (NATA) and National Collegiate Athletic Association (NCAA) have documented that nontraumatic deaths in NCAA football are often linked to exertional conditions, including ER, and average approximately two deaths per season [3]. Sickle cell trait and heat stroke are major contributors to these nontraumatic deaths [3], but ER remains a significant concern, especially during high-intensity workouts with eccentric exercises [4].

In collegiate football, preseason training and scrimmages place considerable strain on athletes. Most cases of ER occur between June and August due to the combined effect of intense exercise, heat illness, and dehydration on overall exertion [2,5], and cases may occur in clusters or team outbreaks [6]. For instance, 13 college football players were hospitalized at one time with ER after an intense off-season workout and most commonly reported bilateral lower extremity muscle pain and swelling, discolored urine, and difficulty walking, underscoring the need for gradual increases in workout volume and intensity, even in athletes [7]. Exercise-induced elevations in muscle damage biomarkers like creatine phosphokinase (CPK), lactate dehydrogenase (LDH), aspartate aminotransferase (AST), and myoglobin can last up to 24 h or more [8], although these elevations are typically transient and part of normal exercise adaptation. Despite this risk, normal reference patterns for key biomarkers following football-specific exertion remain poorly defined. Establishing typical physiological responses to intense exercise and muscle damage extending beyond the measurement of CPK can help to distinguish between routine over-reaching and ER.

CPK is the most widely recognized biomarker for diagnosing ER, as extreme elevations reflect severe muscle breakdown and can signal renal complications [9]. CPK is an enzyme released during muscle breakdown, typically spiking immediately after intense activity and gradually returning to baseline over several days [10]. In Division I football players, mean CPK levels have reached up to 30 times the normal range (5125 IU/L) during two-a-day practices and remained elevated for at least 1 week [8]. Myoglobin and LDH are also biomarkers of cellular stress and muscle damage and tend to follow similar trends to CPK [11,12]. Changes in CPK following a 200 km race were highly correlated with changes in LDH [11], but resistance and aerobic exercise may elicit distinct CPK and LDH responses depending on the stress imposed by the volume and intensity of each type of exercise [13]. Unlike CPK, myoglobin is filtered by the kidneys, and its presence in urine (myoglobinuria) can indicate severe muscle damage and acute kidney injury [14]. Serum myoglobin tends to peak shortly after intense physical activity, and the magnitude of the increase depends on the intensity of the activity, making it useful for early detection of muscle damage and potential complications [12].

The diagnosis of ER may be complicated due to no consistent diagnostic thresholds for CPK (criteria ranging from 1000 to 5000 IU/L), large inter-athlete variability in CPK responses, and higher baseline myoglobin and CPK concentrations in athletes compared to the general population [15]. Kidney and liver biomarkers can help distinguish normal post-exercise adaptations from pathological responses like ER [15]. Alanine aminotransferase (ALT) and AST, liver enzymes, which are also present in skeletal muscle, rise after intense exercise and remain elevated for up to a week or more due to muscle damage, not liver injury [16]. In ER, AST and ALT are elevated in 93% and 75% of the cases, respectively [17], and athletes can experience up to 50-fold increases in AST compared to clinical reference ranges [4,18]. Similarly, blood urea nitrogen (BUN) and creatinine, markers of renal function, are important to monitor as renal impairment can follow severe muscle damage, though the extent of kidney damage varies between individuals [19,20,21]. While transient elevations in liver and kidney enzymes are expected after intense training, marked or persistent increases in combination with other symptoms of ER indicate a pathological process like acute kidney injury rather than normal exercise adaptation.

The present study aimed to characterize post-scrimmage changes in key biomarkers of muscle damage, renal function, and liver stress in collegiate football players to define typical physiological responses to intense exercise. Additionally, correlations between CPK, as the primary biomarker implicated in ER, and other key biomarkers were assessed to determine relationships between muscle damage and physiological function in non-pathological cases of elevated muscle damage. We hypothesized that these biomarkers would acutely increase immediately post-exercise, with partial or complete normalization by 24 h, reflecting transient muscle stress rather than pathological injury.

## 2. Materials and Methods

### 2.1. Participants

Twenty NCAA Division I football players volunteered for the study. Eligibility criteria included being an active member of an NCAA Division I football team and being cleared by a team physician to participate in the fall training camp and associated scrimmages. The study was approved by the Louisiana State University Institutional Review Board (IRB# 4269), and informed consent was obtained from each participant prior to any assessments. Of the 20 football players, 1 player withdrew from the study, and 2 players were later identified by sports medicine staff as having sickle cell trait, which may alter responses to muscle damage [22], and were consequently excluded from this analysis. Therefore, 17 participants were included in the subsequent analysis. None of the players included in the study were diagnosed with ER during their participation. Because biospecimen collection required athletes to present within a tightly controlled immediate post-scrimmage (IPS) time window to ensure standardized sampling, full-team participation was not feasible. Additionally, participation required athletes to report on their day off, which limited overall enrollment. Therefore, this study reflects a voluntary sample from a single site, sport, and competitive year.

### 2.2. Study Design

The present study was a prospective investigation of muscle damage, kidney, and liver biomarker responses following scrimmages in Division I collegiate football players. Primary outcomes of the study included biomarkers of muscle damage, and secondary outcomes included biomarkers of renal function and liver stress, as well as symptoms of ER. The study procedures were scheduled to align with the team’s existing fall training camp calendar and were not designed to alter planned football activities. Whole blood and urine samples were taken immediately (IPS) and 24 h after (24hPS) two separate scrimmages during Fall training camp, separated by 1 week. Scrimmages were full-contact, simulated game sessions conducted under typical preseason conditions. The two scrimmages represented the only scrimmages scheduled during fall camp that season and were each followed by a scheduled team recovery day; therefore, biospecimen collections were aligned with this schedule to capture recovery in a low-load context. During each sample collection, participants also completed a physician exam questionnaire to determine if they were experiencing any symptoms of ER. The questionnaire included 5 yes/no questions (nausea/vomiting, dizziness, cramps, abdominal pain, and headache), frequency of urination (0–3, 4–8, or 8+ times/day), and color of urine (clear, light, dark, or red). If participants reported a headache, they were asked to rate the headache as mild, moderate, or severe.

**Figure 1:**
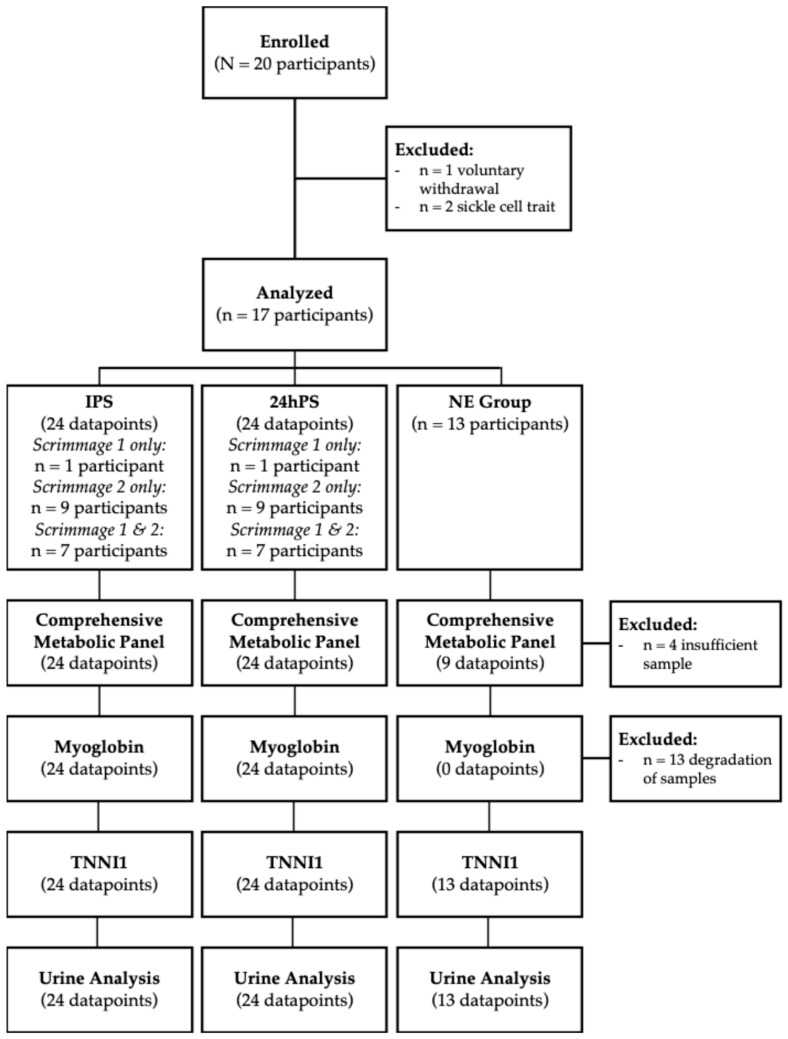
Flow diagram of participant recruitment, exclusion, and analysis for each timepoint and measure. Urine analysis includes urine specific gravity (USG) and color analysis. TNNI1: troponin-I-skeletal-slow-twitch.

A total of 8 players participated in testing after the first scrimmage, and 16 after the second scrimmage. Seven players participated in both scrimmages, for a total of 24 datapoints at each timepoint. Additionally, a subset of 13 players (who participated in 20 out of 24 post-scrimmage data collections) provided blood and urine samples and completed the physician exam questionnaire following a 48 h rest period during the season to act as a non-exertion comparator group (NE); however, metabolic panels could be run on only 9 of the 13 samples. Figure 1 represents the flow of participants recruited and samples analyzed for each timepoint and measure.

### 2.3. Blood and Urine Analysis

Whole blood samples were collected via venipuncture of the antecubital vein into SST vacutainers, centrifuged at 1500× *g* for 12 min, and the resultant serum was aliquoted into cryovials and stored at −80 °C until future analysis. Serum samples were used for a comprehensive, 26-panel metabolic blood test (DxC 700 AU Chemistry Analyzer, Beckham Coulter, Brea, CA, USA), including biomarkers of muscle damage (CPK, LDH), renal function (creatinine, BUN), and liver stress (AST, ALT, total bilirubin). Additional ELISAs were run in accordance with the manufacturer’s instructions to quantify serum myoglobin (Abcam, Cambridge, UK) and troponin-I-skeletal-slow-twitch (TNNI1; MyBioSource, San Diego, CA, USA), also representing biomarkers of muscle damage.

Urine samples were collected, analyzed for urine specific gravity (USG) and color using an 8-color scale [23], aliquoted into cryovials, and stored at −80 °C until all samples could be analyzed for creatinine and myoglobin (Abcam, Cambridge, UK) using ELISAs, in accordance with manufacturer’s instructions. Laboratory staff were blinded to the timepoints and participants when analyzing blood and urine samples. Due to degradation of samples, serum and urine myoglobin could not be quantified for NE samples.

### 2.4. Estimated Glomerular Filtration Rate (eGFR)

Estimated glomerular filtration rate (eGFR) was calculated as a measure of kidney function using the CKD-EPI equation [24] and was adjusted for body surface area (BSA), calculated using the Du Bois and Du Bois equation [25], to account for potential underestimation of eGFR in subjects with a BSA higher than the typical index value (1.73 m^2^) [26].

$$\begin{array}{c} eGFR=142\times {min\;(S_{cr}/\kappa ,1)}^{\alpha }\times {max\;(S_{cr}/\kappa ,1)}^{-1.200}\times 0.9938^{Age\;(y)}\times \frac{BSA}{1.73}\\ BSA=0.007184\times {weight\;(kg)}^{0.425}\times {height\;\left(cm\right)}^{0.725} \end{array}$$

where eGFR = estimated glomerular filtration rate (mL/min), S_cr_ = serum creatinine (mg/dL), κ = 0.9, α = −0.302, and BSA = body surface area (m^2^).

### 2.5. Statistical Analysis

All statistical analyses were conducted using JMP Pro 17 (SAS Institute Inc., Cary, NC, USA). All data are presented as mean ± SD. Initial descriptive data are presented on post-scrimmage biomarker responses relative to reference values to identify biomarkers that may exceed them.

Linear regressions were performed to explore the associations between muscle damage and organ function in football players with elevated muscle damage but not ER. As the primary biomarker used in the diagnosis of ER, CPK was included as the independent variable, and markers of muscle damage (LDH, TNNI1, serum and urine myoglobin), renal function (serum and urine creatinine, BUN, eGFR), and liver stress (AST, ALT, total bilirubin) were used as dependent variables. Pearson’s correlation coefficients (r) and corresponding *p*-values were reported, with statistical significance defined as *p* < 0.05 for all analyses.

To investigate the effect of time on biomarker concentration, a subset of data containing only participants who returned for NE data collection was used (*n* = 13; 20/24 post-scrimmage data collections). A linear mixed-effects model was constructed for each biomarker, with time (NE, IPS, 24hPS) as a fixed effect and participant ID as a random effect to account for individual variation across repeated measures. Where significant main effects were observed (α = 0.05), post hoc analyses were conducted using Student’s *t*-tests.

## 3. Results

### 3.1. Participant Characteristics

Participant characteristics are presented in Table 1. The cohort consisted of 11 backs (wide receiver, safety, linebacker, cornerback, fullback, and tight end) and 6 linemen (center, offensive tackle, offensive line, defensive line, and defensive end). Of the 13 NE samples collected, 3 were from linemen and 10 were from backs. Of the 24 samples collected at each timepoint post-scrimmage, 8 were from linemen and 16 were from backs. Due to the low sample size in the linemen group, no additional positional analysis was conducted. Reference values, NE, IPS, and 24hPS biomarker concentrations for the entire dataset are included in Table 2.

Average USG and urine color were 1.031 ± 0.006 and 5.3 ± 1.4 for IPS, 1.027 ± 0.007 and 4.4 ± 1.9 for 24hPS, and 1.028 ± 0.005 and 4.2 ± 1.8 for the NE comparator group, respectively. None of the athletes reported red urine, and no symptoms were reported in over 95% of the data collection. None of the players included in the study were diagnosed with ER during their participation.

### 3.2. Post-Scrimmage Biomarker Responses

**Figure 2:**
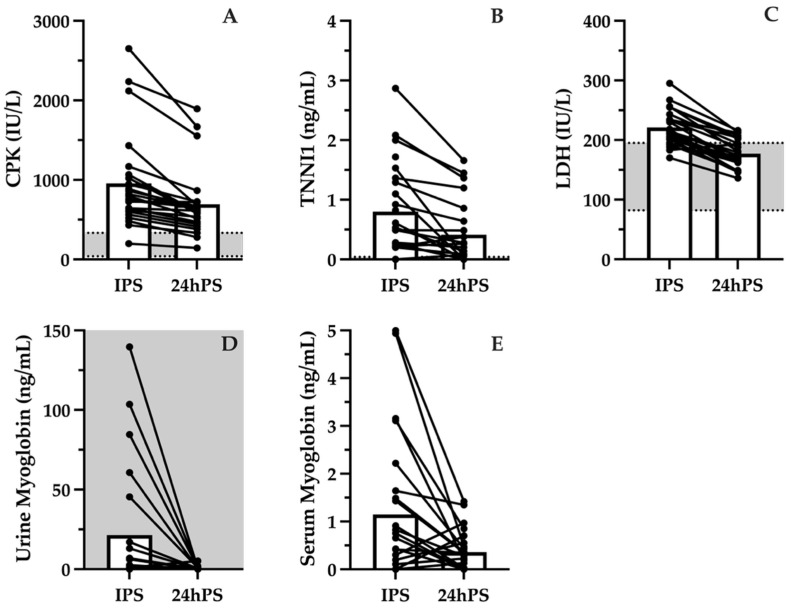
Muscle damage biomarker concentrations, including creatine phosphokinase (CPK; (**A**)), troponin-I-skeletal-slow-twitch (TNNI1; (**B**)), lactate dehydrogenase (LDH; (**C**)), urine myoglobin (**D**), and serum myoglobin (**E**), measured immediately post-scrimmage (IPS) and 24 h post-scrimmage (24hPS) compared to reference values (grey shaded band with dotted line). Bars represent mean values. Points represent individual responses.

Biomarkers of muscle damage demonstrated significant elevations IPS that persisted at 24hPS. Large increases in CPK outside of reference values (38–333 IU/L) were found IPS (955.4 ± 597.2 IU/L), and values decreased but remained elevated above the normal range at 24hPS (693.6 ± 424.7 IU/L; Figure 2A). Similarly, TNNI1 concentrations were greater than reference values (<0.04 ng/mL) at both IPS (0.80 ± 0.78 ng/mL) and 24hPS (0.42 ± 0.54 ng/mL; Figure 2B). Serum LDH measured IPS (221.3 ± 29.8 IU/L) was greater than the reference value (82–195 IU/L) but returned to within the normal range by 24hPS (177.1 ± 23.9 IU/L; Figure 2C). Urine myoglobin also increased outside of reference values (<5 ng/mL) IPS (21.4 ± 38.9 ng/mL) but returned to within normal range by 24hPS (0.5 ± 1.1 ng/mL; Figure 2D). Despite elevations in serum myoglobin IPS (1.15 ± 1.51 ng/mL) compared to 24hPS (0.36 ± 0.42 ng/mL), values remained in the reference range (<72 ng/mL; Figure 2E).

**Figure 3:**
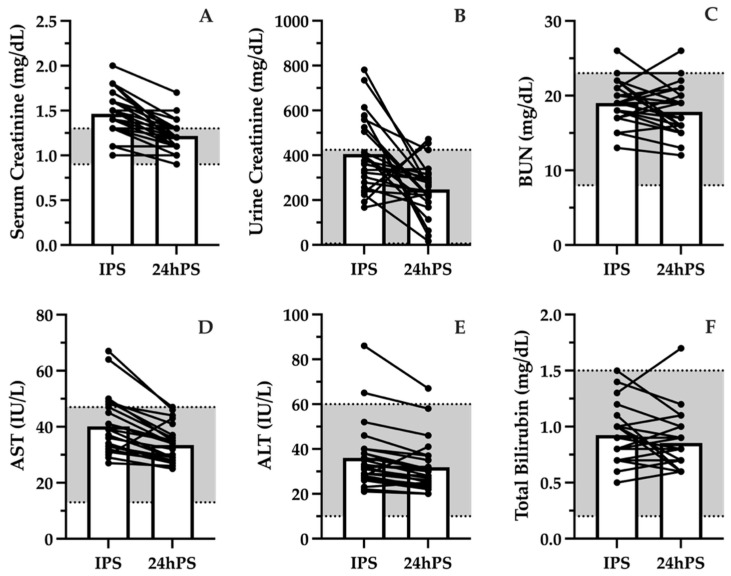
Biomarkers of renal function (serum creatinine (**A**), urine creatinine (**B**), blood urea nitrogen [BUN (**C**)], and liver stress (aspartate aminotransferase [AST (**D**)], alanine aminotransferase [ALT (**E**)], bilirubin (**F**)) immediately post-scrimmage (IPS) and 24 h post-scrimmage (24hPS) compared to reference values (grey shaded band with dotted lines). Bars represent mean values. Points represent individual responses.

Markers of renal function showed modest increases following exercise. Blood creatinine increased IPS (1.46 ± 0.25 mg/dL) but returned to within reference range (0.9–1.3 mg/dL) by 24hPS (1.22 ± 0.18 mg/dL; Figure 3A). Urine creatinine (Figure 3B) and BUN (Figure 3C) remained within reference range at both IPS and 24hPS. Despite remaining within reference range, eGFR was reduced IPS (99.7 ± 24.2 mL/min) compared to 24hPS (123.2 ± 26.8 mL/min).

Markers of hepatic stress remained within reference ranges following the scrimmage, although they were slightly elevated IPS compared to 24hPS. Specifically, AST decreased from 40.2 ± 10.5 IU/L IPS to 33.5 ± 6.6 IU/L 24hPS (Figure 3D), and ALT decreased from 36.0 ± 14.6 IU/L IPS to 31.9 ± 11.6 IU/L 24hPS (Figure 3E). Minimal changes in total bilirubin were noted between the reference range (0.2–1.5 mg/dL) and measurements taken IPS (0.93 ± 0.25 mg/dL) and 24hPS (0.85 ± 0.26 mg/dL; Figure 3F).

### 3.3. Correlations Between CPK (Muscle Damage) and Ancillary Biomarkers of Renal and Hepatic Function

**Figure 4:**
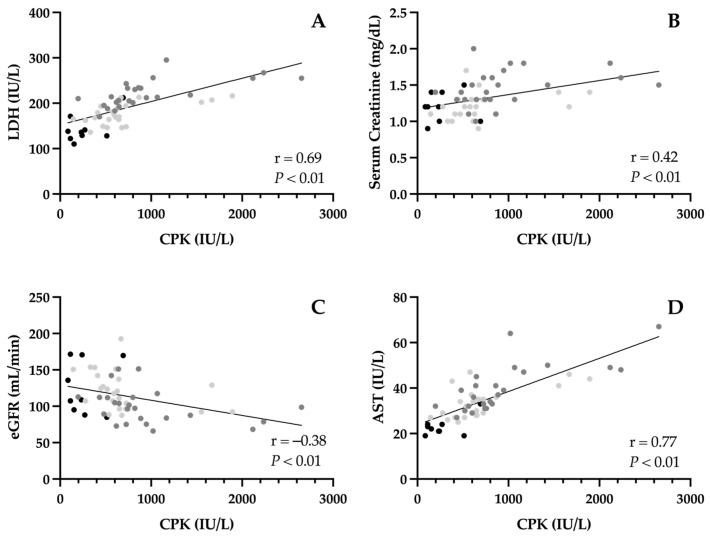
Correlations between creatine phosphokinase (CPK) and lactate dehydrogenase (LDH [muscle damage] (**A**)), serum creatinine (renal function (**B**)), estimated glomerular filtration rate (eGFR [renal function] (**C**)), and aspartate aminotransferase (AST [liver stress] (**D**)). • non-exercise comparator group; • immediately post-scrimmage; • 24 h post-scrimmage.

CPK was positively correlated with LDH (r = 0.69, *p* < 0.01; Figure 4A) and serum myoglobin (r = 0.57, *p* < 0.01). However, no relationship was observed between CPK and urine myoglobin (r = 0.11, *p* = 0.48) or TNNI1 (r = −0.09, *p* = 0.50).

With respect to renal function biomarkers, CPK was positively correlated with serum creatinine (r = 0.42, *p* < 0.01; Figure 4B) and negatively correlated with eGFR (r = −0.38, *p* < 0.01; Figure 4C). Additionally, a non-significant, positive trend was found between CPK and urine creatinine (r = 0.25, *p* = 0.06). No association was observed between CPK and BUN (r = −0.17, *p* = 0.20).

Correlations between CPK and liver stress biomarkers were positive and linear for both AST (r = 0.77, *p* < 0.01; Figure 4D) and ALT (r = 0.38, *p* < 0.01). No relationship was observed between CPK and total bilirubin (r = 0.22, *p* = 0.10).

### 3.4. Effect of Time on Biomarker Recovery

An effect of time was found on CPK and LDH, whereby concentrations were statistically different between all three timepoints (*p* < 0.01 for both). CPK was elevated IPS (991.6 ± 560.8 IU/L) compared to NE (267.7 ± 205.3 IU/L) and started to decline by 24hPS (739.2 ± 442.6 IU/L), although it did not fully recover. The same elevations in LDH were noted from NE (143.0 ± 30.8 IU/L) to IPS (231.3 ± 29.2 IU/L) and 24hPS (183.8 ± 23.6 IU/L). An effect of time on TNNI1 was also found (*p* < 0.01); however, concentrations were greater at both NE (0.62 ± 0.72 ng/mL) and IPS (0.62 ± 0.61 ng/mL) compared to 24hPS (0.27 ± 0.39 ng/mL).

For renal function biomarkers, an effect of time was found for serum creatinine (*p* < 0.01) with concentrations greater IPS (1.51 ± 0.24 mg/dL) compared to NE (1.20 ± 0.21 mg/dL) and 24hPS (1.22 ± 0.16 mg/dL). An effect of time was also found for eGFR (*p* < 0.01), whereby eGFR was lower IPS (94.7 ± 26.4 mL/min) compared to 24hPS (120.8 ± 30.2 mL/min) and NE (125.8 ± 36.8 mL/min). Both urine creatinine (*p* = 0.05) and BUN (*p* = 0.06) trended toward an effect of time. Urine creatinine concentrations were greatest IPS (392.4 ± 176.6 mg/dL) but decreased below NE (270.2 ± 158.9 mg/dL) at 24hPS (267.1 ± 126.3 mg/dL). BUN was also greatest IPS (19.5 ± 2.1 mg/dL) but was lower at NE (17.0 ± 2.0 mg/dL) compared to 24hPS (18.5 ± 3.2 mg/dL).

Lastly, an effect of time was found on all 3 biomarkers of liver stress (*p* ≤ 0.01 for all). Post hoc analyses determined that both AST and ALT concentrations were different at all three timepoints, with concentrations increasing from NE to IPS and not fully recovering by 24hPS. Total bilirubin was greater IPS (0.97 ± 0.26 mg/dL) than at NE (0.72 ± 0.16 mg/dL), but no difference was found between NE and 24hPS (0.86 ± 0.21 mg/dL).

## 4. Discussion

The present study investigated physiological responses of collegiate football players to intense exercise by characterizing post-scrimmage changes in key biomarkers of muscle damage, renal function, and liver stress. The primary findings suggest that most biomarkers of muscle damage exceed reference values and remain elevated above NE values 24 h post-scrimmage in football players not diagnosed with ER. In contrast, other biomarkers of interest, except serum creatinine, largely remain within reference values, despite transient elevations immediately post-scrimmage. Several biomarkers, including AST and ALT, remained elevated 24hPS, suggesting ongoing physiological stress and incomplete recovery in some athletes, and were positively correlated with CPK, indicating a relationship between biomarkers of muscle damage and liver function. Understanding these biomarker fluctuations is crucial for establishing typical physiological responses to intense exercise in non-pathological cases of elevated muscle damage.

Biomarkers of liver and muscle damage, such as CPK, LDH, and AST, exhibited significant elevations IPS, reflecting acute muscle damage and possible liver stress due to strenuous activity. These findings align with Pettersson et al., who reported that AST, ALT, CPK, LDH, and serum myoglobin all increased significantly after a single weightlifting session in healthy, moderately active but weightlifting-naïve men, with liver biomarkers remaining elevated for 7 days post-exercise [16]. The additional recovery time for liver biomarkers to return to normal may be explained by a greater magnitude of muscle damage caused by more intense exercise in less trained individuals, reflected in the exaggerated biomarker response which saw several participants reach CPK values over 48,000 IU/L and serum myoglobin concentrations over 2999 ng/mL [16], compared to an average of 955 IU/L and 1.15 ng/mL in the present study. Since AST and ALT also originate in skeletal muscle, it is possible that the elevations are caused by muscle damage-induced release into the bloodstream. However, the contributions of the liver and skeletal muscle to AST and ALT elevations in the blood cannot be determined from the present analysis. In rhabdomyolysis patients, positive correlations have been identified between CPK and ALT or AST, but not other biomarkers of liver function, including bilirubin and γ-glutamyl transferase, and concentrations of AST may fall in parallel with CPK during recovery [17,27]. Therefore, an increase in ALT or AST, which is proportionate to elevations in CPK, can occur in the absence of liver injury and may be indicative of muscle damage, but additional liver biomarkers that do not have a shared origin with muscle may help to confirm the differentiation between liver and muscle injury [17,27,28]. In combination with the strong correlations observed between CPK, LDH, and AST, these data reinforce their collective role as indicators of muscle stress and the importance of accounting for prior physical activity in the clinical interpretation of biomarkers.

Post-scrimmage measurements of creatinine, BUN, and eGFR suggested minimal, transient renal stress, potentially resulting from dehydration, heat stress, increased muscle breakdown, and heightened metabolic demands [29,30,31]. Serum creatinine rose ~25%, and BUN approached the upper normal limit, which followed similar but less severe trends as previously reported. In marathon runners, 82% developed at least stage 1 acute kidney injury, based on a minimum 1.5-fold increase in serum creatinine, immediately following a marathon, which mostly returned to normal 24 h post-marathon [32]. Elevated biomarkers may not always signify pathology, but the long-term implications for kidney and liver health remain uncertain, underscoring the need for further research to establish clinically significant biomarker thresholds.

A notable and unexpected finding was the presence of elevated TNNI1 in the NE comparator group for most athletes compared to reference values, with concentrations declining at 24hPS. As TNNI1 is specific to slow-twitch skeletal muscle, its pattern reflects muscle strain associated with exercise rather than cardiac stress [33]. Given the elevations observed in the NE group and partial recovery at 24hPS, the presence of TNNI1 may suggest ongoing muscle remodeling or cumulative fatigue from prior training loads. These findings emphasize the need for further investigation into TNNI1 dynamics during repeated bouts of high-intensity activity.

The present study’s strengths include a well-defined elite athletic population, repeated measures design across NE, IPS, and 24hPS timepoints, and a comprehensive biomarker panel assessing muscle damage, renal stress, and liver function. While the study is limited in follow-up to 24hPS, in many collegiate sports, it is uncommon to have 48 h or more of complete rest from exercise between competition or training sessions. Therefore, understanding recovery within a 24 h window may provide more ecologically relevant insights into the characterization of normal responses than altering typical training schedules to allow for research on extended recovery times. While evaluating biomarker responses with repeated loading on consecutive days poses an interesting and novel question, this is beyond the scope of the current research and presents additional logistical challenges with regard to athlete recruitment and compliance. Future research should consider additional assessments to quantify training loads and dehydration and understand individual variability in exercise-induced muscle damage. Additionally, power analysis was not conducted due to expected limitations in recruitment, and some myoglobin data were lost due to degradation of samples.

## 5. Conclusions

In summary, intense exercise induces significant fluctuations in biomarkers related to muscle damage, renal function, and liver stress among collegiate football players. Key findings from this study include positive correlations between CPK and biomarkers of renal stress (serum creatinine) and liver stress (AST, ALT), and the presence of elevated TNNI1 in the NE comparator group, all of which highlight new aspects of muscle, kidney, and liver stress during exercise. By characterizing post-scrimmage changes in key biomarkers, this research enables a more accurate understanding of abnormal responses and aids in preventing unnecessary harm from missed or misinterpreted warning signs. These findings underscore the importance of individualized monitoring of biomarkers to inform training programs, recovery protocols, and clinical assessments. Tracking biomarker trends over time, rather than relying on single measurements, provides a comprehensive understanding of an athlete’s physiological state. Coaches, trainers, and medical staff should prioritize monitoring high-risk athletes, adjusting training loads, and ensuring adequate hydration and nutrition to optimize recovery and prevent overtraining.

## Figures and Tables

**Table 1:** Participant Characteristics.

	Backs (*n* =11)	Line (*n* = 6)	All (N = 17)
Race (*n* [%] Caucasian)	3 [27%]	0 [0%]	3 [18%]
Age (y)	20.5 ± 1.0	20.8 ± 0.8	20.6 ± 0.9
Weight (kg)	101.8 ± 13.3	135.1 ± 9.8	113.6 ± 20.2
Height (m)	1.87 ± 0.04	1.93 ± 0.04	1.89 ± 0.05
BMI (kg/m^2^)	28.9 ± 3.3	36.5 ± 3.6	31.6 ± 5.0
BSA (m^2^)	2.27 ± 0.15	2.62 ± 0.07	2.40 ± 0.21

BMI: body mass index; BSA: body surface area. Data are presented as mean ± SD.

**Table 2:** Biomarker concentrations in the non-exercise (NE) comparator group, immediately post-scrimmage (IPS), and 24 h post-scrimmage (24hPS) compared to reference values.

	Reference	NE	Higher than Reference (*n*/N [%])	IPS	Higher than Reference (*n*/N [%])	24hPS	Higher than Reference (*n*/N [%])
**Muscle Damage**							
CPK (IU/L)	38~333	267.7 ± 205.3	2/9 [22%]	955.4 ± 597.2	23/24 [96%]	693.6 ± 424.7	21/24 [88%]
LDH (IU/L)	82~195	143.0 ± 30.8	1/9 [11%]	221.3 ± 29.8	19/24 [79%]	177.1 ± 23.9	6/24 [25%]
TNNI1 (ng/mL)	<0.04	0.62 ± 0.72	11/13 [85%]	0.80 ± 0.78	20/24 [83%]	0.42 ± 0.54	15/24 [68%]
Serum Myoglobin (ng/mL)	<72.0			1.15 ± 1.51	0/24 [0%]	0.36 ± 0.42	0/24 [0%]
Urine Myoglobin (ng/mL)	<5.0			21.4 ± 38.9	10/23 [43%]	0.5 ± 1.1	1/24 [4%]
**Renal Function**							
Serum Creatinine (mg/dL)	0.9~1.3	1.20 ± 0.21	3/9 [33%]	1.46 ± 0.25	15/24 [63%]	1.22 ± 0.18	4/24 [17%]
Urine Creatinine (mg/dL)	6~424	270.2 ± 158.9	1/9 [11%]	405.6 ± 168.9	8/24 [33%]	248.1 ± 116.1	2/24 [8%]
BUN (mg/dL)	8~23	17.0 ± 2.0	0/9 [0%]	19.0 ± 2.9	1/24 [4%]	17.8 ± 3.3	1/24 [4%]
eGFR (mL/min)	>90	125.9 ± 36.8	2/9 [22%] *	99.7 ± 24.2	10/24 [42%] *	123.2 ± 26.8	2/24 [8%] *
**Liver Function**							
AST (IU/L)	13~47	22.9 ± 4.2	0/9 [0%]	40.2 ± 10.5	6/24 [25%]	33.5 ± 6.6	0/24 [0%]
ALT (IU/L)	10~60	21.8 ± 3.4	0/9 [0%]	36.0 ± 14.6	2/24 [8%]	31.9 ± 11.6	1/24 [4%]
Total Bilirubin (mg/dL)	0.2~1.5	0.72 ± 0.16	0/9 [0%]	0.93 ± 0.25	0/24 [0%]	0.85 ± 0.26	1/24 [4%]

CPK: creatine phosphokinase; LDH: lactate dehydrogenase; TNNI1: troponin-I-skeletal-slow-twitch; BUN: blood urea nitrogen; eGFR: estimated glomerular filtration rate; AST: aspartate aminotransferase; ALT: alanine aminotransferase. NE, IPS, and 24hPS data are presented as mean ± SD. Higher than reference value data are calculated at each timepoint, with the exception of eGFR, which represents lower than reference values (denoted by *), and presented as *n*/N [%], where *n* = number of samples above reference value, N = total number of samples, % = percentage of samples above reference value. Except for eGFR, none of the values were below reference values.

## Data Availability

The data contained in this manuscript are available upon request to the corresponding author.

## Abbreviations

The following abbreviations are used in this manuscript:

ER	Exertional Rhabdomyolysis
NCAA	National Collegiate Athletic Association
CPK	Creatine Phosphokinase
LDH	Lactate Dehydrogenase
AST	Aspartate Aminotransferase
ALT	Alanine Aminotransferase
BUN	Blood Urea Nitrogen
IPS	Immediately Post-Scrimmage
24hPS	24-Hours Post-Scrimmage
NE	Non-Exertion Comparator Group
TNNI1	Troponin-I-Skeletal-Slow-Twitch
eGFR	Estimated Glomerular Filtration Rate
